# A case of 3 months old Japanese boy with sporadic congenital none-autoimmune hyperthyroidism

**DOI:** 10.1186/1687-9856-2015-S1-P98

**Published:** 2015-04-28

**Authors:** Saori Kinjo, Masayuki Kanno, Kei Matayoshi, Kiyotaka Takefuta, Ayako Izumi, Chiho Sugisawa, Satoshi Narumi, Moriyasu Kohama

**Affiliations:** 1 Department of Pediatrics, Okinawa Chubu Hospital, Miyazato, Uruma, Okinawa Prefecture, Japan; 2 Department of Naonatology, Okinawa Chubu Hospital, Japan; 3 Department of Endocrine & Metabolism, Showa University Fujigaoka Hospital, Japan; 4 Department of Pediatrics, Keio University, Japan

## 

Sporadic congenital none-autoimmune hyperthyroidism is rare disease. It causes the gain of function with TSH receptor gene mutation. We report 3 months old Japanese boy with sporadic congenital none-autoimmune hyperthyroidism. Our case was born in the 34th week of gestation with a birth weight 1830g, his length 42.0cm, his head circumference 29.5 cm as low birth weight baby between non consanguineous parents. Oligohydramnios was pointed out his perinatal period. There was no history of thyroid disease in other family members. He showed failure to thrive after the age of one month in spite of increasing nutrition. At the age of 3 months, His weight was 3.5kg, his length 54.6cm. He presented with tachycardia (170~180/min), severe sweating, mild exophthalmos and no goiter. We examined carefully and found he was suffered from hyperthyroidism. The thyroid function showed TSH<0.005 μIU/ml, fT3 20.55 pg/ml, fT4 7.43 ng/dl, Tg603 ng/ml with tests for anti-thyroid antibodies negative. His thyroid ultrasonography showed enlarged as his age, 99mTcO4- scintiscan of thyroid gland showed a homogeneous uptake. His bone age was advanced to 2 years old. Brain MRI showed normal image for his age. His mother had no goiter and had not showed symptoms of hyperthyroidism. Tests for anti-thyroid antibodies were negative with her. We detected TSH receptor gene mutation with our case. The mutation is heterozygous and shows c842G>A,p.Ser281Asn that had been reported before. The boy was treated with amount of 0.8 mg/kg/day of methimazole, his irritability disappears and his heart rate is down. Now his weight gains slowly and his development is appropriate for his age. We conclude that careful examination and follow up need for his development and growth because the severity of sporadic congenital none-autoimmune hyperthyroidism is variety and most reported case were recurrent.

Written informed consent was obtained from the patient's parent or guardian for publication of this abstract and any accompanying images. A copy of the written consent is available for review by the Editor of this journal.

